# Long Short-Term Memory–GPT-4 Integration for Interpretable Biomedical Signal Classification: Proof-of-Concept Study

**DOI:** 10.2196/87962

**Published:** 2026-03-20

**Authors:** Kapil Kumar Reddy Poreddy, Ajit Sahu, Sanjoy Mukherjee, Bhavan Kumar Basavaraju

**Affiliations:** 1 Institute of Electrical and Electronics Engineers, 2962 Millbridge Dr, San Ramon, CA, 94583, United States, 1 5104614814

**Keywords:** artificial intelligence, cloud-based diagnostics, biomedical signal analysis, LSTM networks, long short-term memory, GPT-2, explainable artificial intelligence, health care accessibility, remote health monitoring, physiological data interpretation

## Abstract

**Background:**

Approximately 3.8 billion people lack access to essential health services, and diagnostic interpretation remains a major bottleneck in remote and resource-constrained settings. Limited access to specialists and the complexity of biomedical signal interpretation (eg, electrocardiogram [ECG] and electroencephalogram) contribute to delays in recognizing cardiovascular and neurological conditions.

**Objective:**

The study aimed to develop and evaluate a technical framework integrating long short-term memory (LSTM) networks with GPT-4 to provide automated biomedical signal classification and human-readable interpretations, suitable as a foundation for future deployment in resource-constrained environments.

**Methods:**

The 2-layer LSTM architecture (128→64 units) was selected based on preliminary experiments comparing configurations ranging from single-layer networks (64, 128 units) to deeper architectures (128→64→32 units). The chosen configuration balanced model capacity against overfitting risk and computational efficiency. The framework was evaluated using public PhysioNet datasets: Massachusetts Institute of Technology–Beth Israel Hospital (MIT-BIH) Arrhythmia, Physikalisch-Technische Bundesanstalt (PTB) Diagnostic ECG, Physikalisch-Technischen Bundesanstalt-extra large, Chapman-Shaoxing, Medical Information Mart for Intensive Care-III Waveforms, and Sleep-European data format. A patient-level split protocol (70/15/15) was used to reduce leakage risk. The LSTM architecture (128→64 units) performed temporal feature extraction with softmax-based classification for mutually exclusive classes. GPT-4 was integrated via an application programming interface with structured prompts to generate clinical interpretations from model outputs.

**Results:**

For the expert evaluation, we randomly sampled 50 test cases per dataset (150 total: 30 from each class for MIT-BIH, 25 per class for PTB, and 20 per class for Children's Hospital Boston-Massachusetts Institute of Technology), ensuring balanced class representation. Three board-certified physicians (2 cardiologists for ECG datasets and 1 neurologist for the electroencephalogram dataset) independently reviewed GPT-4–generated interpretations. Reviewers were blinded to whether signals were correctly or incorrectly classified by the LSTM model. Each interpretation was rated on a 5-point Likert scale (1=clinically inappropriate and 5=highly accurate and clinically useful). Interrater reliability was assessed using Fleiss κ (0.78, substantial agreement). On held-out test sets, classification performance was as follows: MIT-BIH 92.3% accuracy (*F*_1_=0.91, AUC=0.95), PTB Diagnostic 94.7% (*F*_1_=0.94, AUC=0.97), Physikalisch-Technischen Bundesanstalt-extra large 88.9% (*F*_1_=0.88, AUC=0.93), Chapman-Shaoxing 91.2% (*F*_1_=0.90, AUC=0.94), Medical Information Mart for Intensive Care-III 89.5% (*F*_1_=0.89, AUC=0.92), and Sleep-European data format 87.3% (*F*_1_=0.86, AUC=0.91). Expert evaluation of generated interpretations (3 board-certified cardiologists) rated clinical accuracy 4.3 out of 5, clarity 4.6 out of 5, and actionability 4.2 out of 5, with strong interrater agreement (κ>0.85).

**Conclusions:**

This proof-of-concept demonstrates an explicit methodological integration of deep learning–based biomedical signal classification with GPT-4–based interpretation, provides a technical foundation for future prospective clinical validation, field studies, and regulatory review prior to clinical deployment in underserved settings.

## Introduction

### Background

Universal health coverage remains limited by uneven access to diagnostic expertise, particularly in remote and resource-constrained environments [1]. In many regions, diagnostic devices may be available, but the trained personnel required to interpret complex biomedical signals are scarce [2]. For cardiovascular and neurological conditions, delays in interpreting diagnostics often lead to deferred treatment and poorer patient outcomes [3].

Recent advances in artificial intelligence (AI) offer an opportunity to assist frontline providers by automating pattern recognition in biomedical signals [4]. Traditional signal processing pipelines depend on hand-crafted features and careful parameter tuning; while foundational, these approaches are often brittle in the presence of noise, artifacts, and inter-patient variability [4]. Deep learning models, including convolutional and recurrent neural networks, have demonstrated improved performance across electrocardiogram (ECG) and electroencephalogram (EEG) classification tasks [4]. However, these systems typically generate technical outputs that are difficult for nonspecialist health care workers to interpret, creating a barrier to adoption in settings where clinical clarity and interpretability are essential [5].

Large language models (LLMs) have recently shown promise in generating readable summaries and decision-support messages for complex technical outputs [5]. However, applying generative models in diagnostic contexts introduces additional risks, including variable outputs, potential hallucination, and the need for careful framing to ensure that generated explanations function as decision support rather than autonomous diagnosis.

This work presents a technical proof-of-concept integrating a long short-term memory (LSTM)-based classifier with GPT-4 to generate structured, clinically oriented interpretations of biomedical signal classifications. The objective is to provide methodological transparency and a reproducible baseline that may support future clinical translation and deployment studies.

- Primary objective: Can an LSTM–GPT-4 pipeline provide accurate biomedical signal classification together with interpretable clinical reasoning suitable for nonspecialist diagnostic support?

- Research questions: Can modality-adaptive preprocessing enable consistent modeling across ECG and EEG signals? Does single-lead selection preserve diagnostic performance while aligning with point-of-care device constraints? Can GPT-4 generate clinically accurate, clear, and actionable interpretations from model outputs?

- Important clarification: This system has not been deployed clinically and is presented strictly as a proof-of-concept. Any clinical deployment would require prospective validation, workflow integration studies, and regulatory review [3,6].

### Contributions

This work contributes to AI-assisted diagnostics research in the following ways:

1. *Modality-specific preprocessing*: Adaptive bandpass filtering (ECG: 0.5‐50 Hz and EEG: 0.5‐30 Hz) for preserving diagnostically relevant features while removing modality-specific artifacts
2. *Single-lead selection strategy*: Explicit methodology ensuring consistent input dimensionality (batch, 3000, 1) and alignment with point-of-care devices
3. *Single-label formulation*: Clear focus on mutually exclusive classification tasks with softmax activation, making it appropriate for primary diagnosis in resource-limited settings
4. *Patient-level data splitting*: Rigorous prevention of data leakage with formal verification (Algorithm 1), ensuring true generalization performance
5. *GPT-4 integration*: Natural language interpretation achieving high clinical accuracy (4.3/5.0) and clarity (4.6/5.0) from expert reviewers
6. *Open-source implementation*: Publicly available codebase with Representational State Transfer application programming interface (API) for community validation and extension
7. *Methodological transparency*: Comprehensive documentation of preprocessing parameters, architecture specifications, training procedures, and evaluation protocols enabling reproducibility

### Related Work

#### Traditional Signal Processing Approaches

Early automated biomedical signal analysis relied on deterministic signal processing techniques, including Fourier transforms, wavelet decomposition, and rule-based feature extraction. In ECG analysis, classical pipelines typically involve beat segmentation followed by handcrafted features such as RR intervals, QRS width, and frequency-domain descriptors [7,8]. While effective in controlled settings, these approaches are sensitive to noise, motion artifacts, and interpatient variability, limiting robustness in real-world deployments [8].

In EEG analysis, spectral methods and rule-based heuristics have been used for sleep stage classification and neurological monitoring. Although clinically established, such approaches require careful parameter tuning and struggle with ambiguous transitions between physiological states, contributing to variability and reduced reliability in automated settings [9].

#### Deep Learning for Biomedical Signal Classification

Deep learning models have demonstrated significant improvements over traditional methods for biomedical time-series classification. Convolutional and recurrent neural networks have been successfully applied to ECG arrhythmia detection, achieving strong performance across multiple benchmark datasets [7,10-12]. LSTM architectures are particularly effective at modeling temporal dependencies in physiological signals, making them well suited for ECG and EEG classification tasks [10-12].

Recent studies have explored attention mechanisms and interpretable deep learning architectures, particularly for multilead ECG analysis [13]. While these models improve feature attribution and performance, they typically require multilead inputs and substantial computational resources, limiting compatibility with portable point-of-care devices commonly used in resource-limited settings [14]. Performance metrics represent mean (SD) across 5-fold cross-validation. The 95% CIs for accuracy were as follows: Massachusetts Institute of Technology-Beth Israel Hospital (MIT-BIH) (91.1%-93.5%), Physikalisch-Technische Bundesanstalt (PTB) (88.9%-92.1%), Children’s Hospital Boston-Massachusetts Institute of Technology (CHB-MIT) (85.2%-88.8%).

Despite strong predictive performance, most deep learning classifiers output numerical labels or probabilities without providing clinically meaningful explanations, which hinders adoption by nonspecialist health care workers [15].

#### Large Language Models and Clinical Interpretability

LLMs have recently shown promise in health care apps, including clinical summarization, medical question answering, and decision support [16,17]. Studies demonstrate that modern LLMs encode substantial clinical knowledge and can generate coherent, human-readable explanations when appropriately constrained [17].

However, deploying generative models in diagnostic contexts introduces additional challenges, including output variability, uncertainty communication, and the risk of overreliance on automated reasoning [18]. Prior work emphasizes that LLMs should function as decision support tools rather than autonomous diagnostic agents, with careful framing and human oversight [15,18].

While LLMs have been integrated with imaging pipelines, particularly in radiology, their application to time-series biomedical signals remains limited. Bridging this gap requires structured integration between signal classifiers and language models, along with methodological transparency and clear limitations [14,16].

#### Synthesis of Prior Work and Research Gaps

Table 1 summarizes representative approaches across biomedical signal processing, deep learning, and health care AI systems.

**Table 1.** Comparison of representative biomedical signal analysis approaches.

Approach	Signal processing	Cross-modal support	Interpretability	Deployment feasibility
Traditional ML^a^ (SVM^b^, RF^c^) [7,8]	Handcrafted	Limited	Moderate	High
LSTM^d^-based ECG^e^ models [10-12]	Learned	Single modal	Low	Moderate
Interpretable DL^f^ (12-lead ECG) [13]	Learned	Single modal	Moderate	Low
LLM^g^-only clinical models [17]	N/A^h^	Text-only	High	High
IoT^i^ fog-enabled systems	N/A	Yes	Low	High
IoMT^j^ security frameworks []	N/A	Yes	Low	Moderate
ML health care reviews	Survey-based	Yes	Moderate	Survey
Telemedicine systems [19,20]	N/A	Yes	Low	High
Proposed framework	Modality-adaptive	ECG + EEG^k^	High	Moderate^l^

a ML: machine learning.

b SVM: support vector machine.

c RF: random forest.

d LSTM: long short-term memory.

e ECG: electrocardiogram.

f DL: deep learning.

g LLM: large language model.

h N/A: not applicable.

i IoT: Internet of Things.

j IoMT: Internet of Medical Things.

k EEG: electroencephalogram.

l Pending prospective clinical validation.

### Identified Research Gaps

The research gaps are as follows:

- Generic preprocessing limits cross-modal robustness: Most deep learning pipelines apply uniform preprocessing across modalities, despite fundamental differences between ECG and EEG signal characteristics [7,9].

- Lack of interpretable outputs for nonspecialists: Existing classifiers provide technical output without contextual explanation, limiting usability for frontline health care workers [15,18].

- Mismatch with point-of-care constraints: Many state-of-the-art models depend on multilead recordings or high-end infrastructure, reducing feasibility in resource-constrained environments [14,19].

## Methods

### Study Design Overview

This is a retrospective technical proof-of-concept study using publicly available biomedical signal datasets. The analytical workflow consisted of (1) dataset acquisition and preprocessing, (2) patient-level data splitting to prevent leakage, (3) LSTM model training and validation using 5-fold cross-validation, (4) classification performance evaluation on held-out test sets, and (5) qualitative expert assessment of GPT-4 generated interpretations. Primary outcomes were classification performance metrics (accuracy, *F*_1_-score, AUC-ROC) on 3 public datasets (MIT-BIH Arrhythmia, PTB Diagnostic ECG, CHB-MIT Scalp EEG). Secondary outcomes included feasibility (computational requirements and processing time) and acceptability (expert clinician ratings of interpretation quality on a 5-point Likert scale, with scores≥4 indicating acceptable quality). All preprocessing, model training, and evaluation code are available in the public GitHub repository to ensure full reproducibility.

### Study Design, Data Flow, and Outcome Measures

This study presents a technical proof-of-concept for automated biomedical signal classification combined with natural language generation for clinician-facing interpretation. The pipeline consists of (1) modality-specific preprocessing and segmentation, (2) deep learning-based classification using recurrent neural networks, and (3) large language model-assisted generation of structured interpretations from model outputs. The evaluation was conducted offline using publicly available datasets.

### Data Sources and Dataset Selection

Public biomedical signal datasets were used to evaluate performance across multiple cardiac and neurological tasks. The included datasets represented common ECG diagnostic benchmarks and EEG sleep staging benchmarks, supporting reproducibility and comparability with prior work in biomedical signal modeling.

### Data Preprocessing

#### Modality-Specific Bandpass Filtering

To preserve diagnostically relevant signal content while reducing baseline drift and noise, the following modality-specific bandpass filtering was applied.

- *ECG filtering:* 0.5‐50 Hz bandpass filtering was used to preserve QRS morphology and reduce baseline wander and high-frequency artifacts commonly present in ambulatory and intensive care unit (ICU) monitoring ECG data.
- *EEG filtering:* 0.5‐30 Hz bandpass filtering was applied to retain physiologically relevant sleep frequency bands while attenuating high-frequency muscle noise.

Filtering was performed using a 5th-order Butterworth filter. Detailed modality-specific filter parameters are summarized in Table 2.

**Table 2.** Modality-specific bandpass filter parameters.

Dataset type	Low cutoff (Hz)	High cutoff (Hz)	Rationale
ECG^a^ datasets	0.5	50	Preserve QRS morphology; reduce baseline drift and noise
EEG^b^ datasets	0.5	30	Preserve sleep-related frequency bands; reduce EMG^c^ artifacts

a ECG: electrocardiogram.

b EEG: electroencephalogram.

c EMG: electromyography.

#### Signal Normalization

To reduce amplitude variability across recordings and improve training stability, *z* score normalization was performed at the segment level:


(1) $$x_{\text{norm}}=\frac{x-\mu }{\sigma }$$

where, μ and σ are the mean and SD computed per segment.

#### Sampling Rate Handling and Segmentation

Signals were segmented using fixed-sample windows to standardize input dimensionality while avoiding interpolation artifacts that may distort diagnostic morphology. Each signal was divided into 3000-sample windows with 50% (1500/3000) overlap, improving coverage near transitions and reducing boundary effects. For ECG datasets, this window size was chosen to capture multiple cardiac cycles for rhythm characterization. For EEG datasets, 3000-sample windows supported epoch-based sleep staging while maintaining a uniform input shape for the neural network. The dataset-specific segmentation strategy is summarized in Table 3.

**Table 3.** Dataset-specific segmentation strategy.

Dataset type	Sampling rate (Hz)	Window size (samples)	Duration (s)	Rationale
ECG^a^ datasets	Dataset-dependent	3000	Rate-dependent	Capture multiple cardiac cycles for rhythm characterization
EEG^b^ datasets	Dataset-dependent	3000	Rate-dependent	Support epoch-based staging while maintaining uniform input shape

a ECG: electrocardiogram.

b EEG: electroencephalogram.

#### Multilead Handling and Lead Selection

To ensure consistent input dimensions and support feasibility for point-of-care devices, a single-lead approach was used when datasets provided multiple leads. The selected lead followed dataset conventions or common diagnostic practice used in prior arrhythmia or sleep modeling pipelines. For ECG datasets such as MIT-BIH, the MLII (lead II) channel was chosen when available, reflecting standard practice in arrhythmia modeling and benchmark comparisons. EEG sleep staging datasets used the primary EEG channel, typically Fpz-Cz, consistent with single-channel sleep staging studies. For ICU waveform datasets with variable lead configurations, the primary available monitoring lead was selected to align with outputs from clinical monitoring devices. The complete lead selection strategy is summarized in Table 4.

**Table 4.** Lead selection strategy.

Dataset	Total leads	Selected lead (example)	Rationale
ECG^a^ datasets (eg, MIT-BIH)^b^	≥2	MLII^c^ or Lead II (if available)	Commonly used in arrhythmia modeling and benchmark comparisons
EEG^d^ Sleep Staging datasets	≥1	Primary EEG channel (eg, Fpz-Cz when available)	Frequently used in single-channel sleep staging studies
ICU^e^ Waveform datasets	Variable	Primary available monitoring lead	Aligns with monitoring device outputs in clinical settings

a ECG: electrocardiogram.

b MIT-BIH: Massachusetts Institute of Technology-Beth Israel Hospital.

c MLII: modified lead II.

d EEG: electroencephalogram.

e ICU: intensive care unit.

#### Dataset Categorization and Label Formulation

The framework was evaluated on 6 PhysioNet datasets, which were categorized according to their label structure. *Single-label datasets*, where each sample belongs to exactly 1 class, included MIT-BIH arrhythmia (5 classes: normal, premature ventricular complex, fusion, atrial premature, and right bundle branch block), Sleep-European data format (EDF) (5 classes: Wake, N1, N2, N3, and rapid eye movement), and Medical Information Mart for Intensive Care (MIMIC)-III Waveforms (4 classes: normal sinus rhythm, atrial fibrillation, ventricular tachycardia, bradycardia). In contrast, *multilabel datasets*, where samples may exhibit co-occurring diagnoses, included PTB Diagnostic ECG (2 classes), Physikalisch-Technischen Bundesanstalt-extra large (PTB-XL) (71 diagnostic statements), and Chapman-Shaoxing (4 rhythm categories). For the single-label datasets, a softmax activation function was applied to the output layer to produce mutually exclusive class probabilities, whereas multilabel datasets used a sigmoid activation per class, allowing independent probability estimates for each diagnostic label.

### Ethical Considerations

#### Ethics Approval and Informed Consent

This study exclusively used publicly available, deidentified biomedical signal datasets obtained from PhysioNet and did not involve direct human subjects research, prospective data collection, or clinical deployment. Institutional Review Board (IRB) approval was not required for this technical proof-of-concept study, as it constitutes a secondary analysis of publicly available, anonymized datasets that are exempt from human subjects review under 45 Code of Federal Regulation 46.104(d)(4). An *institutional case number* was not applicable (secondary analysis of publicly available deidentified data; exempt from IRB review per 45 Code of Federal Regulations 46.104[d][4]).

#### Dataset Ethics and Deidentification

All datasets used in this research were obtained from PhysioNet (physionet.org), a repository that provides ethically approved, deidentified physiological data for research purposes. The MIT-BIH Arrhythmia database was originally collected under protocols approved by the institutional committees of Beth Israel Hospital and the Massachusetts Institute of Technology, and all records are fully deidentified with no patient identifiers retained [21]. The Sleep-EDF Database was collected under ethics approval from the medical ethics committee of the hospital where recordings were performed, and all patient identifiers were removed prior to public release [22]. The MIMIC-III Waveform Database received approval from the IRBs of Beth Israel Deaconess Medical Center (Boston, MA) and the Massachusetts Institute of Technology (Cambridge, MA), and all protected health information was deidentified in accordance with Health Insurance Portability and Accountability Act (HIPAA) standards [23].

#### Algorithmic Fairness and Bias Mitigation

Potential biases inherent in the training datasets are acknowledged. The MIT-BIH Arrhythmia Database was collected during the 1970s-1980s from a predominantly nondiverse patient population at Beth Israel Hospital. The MIMIC-III database, although more recent, reflected the demographic distribution of intensive care unit patients from a single academic medical center in Boston. To address these limitations, dataset demographics and representation gaps were explicitly documented to promote transparency. A single-lead signal analysis approach was adopted to avoid compounding biases that may arise from multilead fusion algorithms performing inconsistently across demographic groups. Patient-level data splitting was used to prevent overfitting to individual subjects; however, population-level biases remain. A critical limitation is that the models have not been validated across diverse racial, ethnic, age, or geographic populations. Any clinical deployment would require extensive fairness audits and validation across underrepresented groups to ensure equitable diagnostic performance.

#### PhysioNet Data Use Agreement

Access to and use of PhysioNet datasets complied with the PhysioNet Credentialed Health Data Use Agreement, which mandates that data be used exclusively for research purposes, prohibits attempts at reidentification, requires proper citation of original data sources, and enforces responsible data handling practices.

#### AI Explainability and Clinical Accountability

The integration of GPT-4 for natural language interpretation introduces important considerations related to explainability and accountability. While LSTM model predictions were deterministic, GPT-4 outputs were probabilistic and may vary between runs. Structured prompt templates were used to promote consistency, although residual variability was acknowledged. The system was explicitly designed as a clinical decision support tool rather than an autonomous diagnostic system, and all outputs included disclaimers requiring review by a qualified health care professional. GPT-4 generated interpretations explicitly stated that AI-assisted outputs must be reviewed by a clinician. In cases where GPT-4 API calls failed, the system provided template-based responses derived from a curated medical knowledge base to ensure continuity of operation without compromising safety.

#### Responsible AI Deployment Framework

This work represented a technical proof-of-concept and has not been deployed in clinical settings or received regulatory clearance for diagnostic use. Translation to clinical practice would require regulatory approval, such as Food and Drug Administration 510(k) clearance or equivalent; prospective multisite clinical validation with IRB approval; fairness auditing across diverse demographic groups; integration into HIPAA-compliant clinical infrastructure with electronic health record support; structured health care worker training programs; and implementation of postdeployment monitoring systems to detect performance drift.

#### Equity and Access Considerations

Although the system was intended to support health care delivery in underserved communities, several equity-related challenges were recognized. Cloud-based deployment requires reliable internet connectivity, which may limit accessibility in remote settings. GPT-4 API costs may present financial barriers in resource-constrained environments, indicating the need for cost-optimized alternatives or subsidized access models. Current system outputs are limited to English, necessitating multilingual support for equitable deployment. Additionally, the system assumes a baseline level of clinical training among users, underscoring the importance of accompanying education and training initiatives.

#### Commitment to Responsible Research

To promote transparency and enable independent ethical review, all source code is publicly available under an open-source license [24], and model architectures, training procedures, dataset selection criteria, and known limitations are fully documented. Performance metrics reported both strengths and failure modes. Any future clinical implementation will require prospective IRB approval with informed consent protocols, algorithmic impact assessments aligned with emerging AI governance frameworks, engagement with underserved communities, and continuous monitoring for algorithmic bias and performance degradation.

#### Future Ethical Considerations for Clinical Translation

Should this system advance toward clinical implementation, several ethical requirements must be addressed, including full IRB review and approval for prospective validation studies, informed consent protocols for AI-assisted diagnostic interpretation, validation across diverse demographic groups to ensure algorithmic fairness, implementation of human-in-the-loop clinical oversight, deployment within HIPAA-compliant data infrastructure, and attainment of regulatory clearance in target deployment regions.

### Deep Learning Model

#### Model Architecture

A recurrent neural network classifier was implemented using a 2-layer LSTM backbone to capture temporal dependencies in biomedical time-series signals, consistent with prior ECG classification work. The first LSTM layer contains 128 units with return sequences enabled, followed by a dropout layer of 0.2. The second LSTM layer has 64 units, followed by another dropout layer of 0.2. A dense layer with 32 units and rectified linear unit (ReLU) activation precedes the output layer, which uses a softmax or sigmoid activation depending on the classification task. The complete neural network architecture is summarized in Table 5.

**Table 5.** Neural network architecture.

Layer	Output shape	Activation
Input^a^	(None, 3000, 1)	—^b^
LSTM^c^-1 (128 units, return sequences)	(None, 3000, 128)	Tanh
Dropout (0.2)	(None, 3000, 128)	—
LSTM-2 (64 units)	(None, 64)	Tanh
Dropout (0.2)	(None, 64)	—
Dense (32 units)	(None, 32)	ReLU^d^
Output layer^e^	(None, C)	Softmax or sigmoid

a Input: (batch, 3000, 1).

b Not applicable.

c LSTM: long short-term memory.

d ReLU: rectified linear unit.

e Output: dataset-dependent class probabilities.

#### Output Formulation (Single-Label vs Multilabel)

##### Single-Label Classification (Mutually Exclusive Classes)

For tasks where each segment belongs to exactly 1 class, softmax activation was used:


(2) $${\hat{y}}_{c}=\frac{e^{z_{c}}}{\sum_{j=1}^{C}e^{z_{j}}},\sum_{c=1}^{C}{\hat{y}}_{c}=1$$

##### Multilabel Classification (Co-Occurring Diagnoses)

For tasks where segments may contain multiple labels (eg, ECG diagnostic statements), sigmoid activation was used per class:


(3) $${\hat{y}}_{c}=\sigma (z_{c})=\frac{1}{1+e^{-z_{c}}}$$

This enables independent per-class probabilities and supports multilabel evaluation protocols.

### Training Configuration

The model was trained using the Adam optimizer with standard regularization and early adaptation to class imbalance via weighted loss. Architectural parameters included 2 LSTM layers with 128 and 64 units, respectively, followed by a dense layer with 32 units and dropout of 0.2. ReLU activation was applied in hidden layers, and softmax was used in the output layer. Optimization used a learning rate of 0.001 with β₁=.9, β₂=.999, and *ε*=1×10⁻⁷. Training was performed over 50 epochs with a batch size of 32, using categorical cross-entropy as the loss function. Regularization strategies included class weighting (inversely proportional to class frequency) and a ReduceLROnPlateau scheduler with a reduction factor of 0.5 and patience of 10 epochs. Data augmentation was applied with a probability of 0.5, including Gaussian noise (factor 0.05), amplitude scaling (0.8-1.2), and temporal shifts (10%). Detailed training hyperparameters are summarized in Table 6.

**Table 6.** Training hyperparameters.

Category and parameter	Value
Architecture	
LSTM^a^ layer 1 units	128
LSTM layer 2 units	64
Dropout rate	0.2
Dense layer units	32
Activation (hidden)	ReLU^b^
Activation (output)	Softmax
Optimization	
Optimizer	Adam
Learning rate	0.001
β₁	0.9
β₂	0.999
ε	1×10^-7^
Batch size	32
Epochs	50
Loss function	Categorical cross-entropy
Regularization	
Class weighting	Balanced (inversely proportional)
Learning rate scheduler	ReduceLROnPlateau
Learning rate reduction factor	0.5
Learning rate patience	10 epochs
Data augmentation	
Augmentation probability	0.5
Noise factor (Gaussian)	0.05
Amplitude scaling range	(0.8-1.2)
Temporal shift ratio	10%
Loss function	Categorical cross-entropy

a LSTM: long short-term memory.

b ReLU: rectified linear unit.

### Data Splitting Strategy: Patient-Level Splitting to Prevent Leakage

To reduce the risk of inflated performance due to segment overlap across splits, patient-level splitting was applied when patient identifiers were available. All segments derived from a given patient were assigned exclusively to train, validation, or test partitions. This approach aligns with methodological guidance emphasizing the importance of proper separation between training and evaluation data in medical AI and broader concerns about reproducibility in AI systems (Textbox 1).

Textbox 1. **Patient-level data splitting.**

**Input:** dataset D with patient IDs P, samples X, labels Y

**Parameters:** split ratios $r_{\text{train}}=0.70,r_{\text{val}}=0.15,r_{\text{test}}=0.15,\text{seed}=42$

**Output:** $\left(X_{\text{train}},Y_{\text{train}}),(X_{\text{val}},Y_{\text{val}}),(X_{\text{test}},Y_{\text{test}}\right)$

1. Extract unique patient IDs *P* from *D*
2. Shuffle *P* using fixed seed
3. Assign the first 70% of patients to train, next 15% to validation, remaining 15% to test
4. Extract all segments belonging to patients in each partition
5. Verify no patient overlap:

$P_{\text{train}}\cap P_{\text{val}}=\emptyset ,P_{\text{train}}\cap P_{\text{test}}=\emptyset ,P_{\text{val}}\cap P_{\text{test}}=\emptyset$

### Data Augmentation

To reduce overfitting and improve robustness under physiologic variability and sensor noise, augmentation was applied only to training segments. Augmentations included additive Gaussian noise, amplitude scaling, and temporal shifting, consistent with common practices in ECG modeling. Augmentations were applied probabilistically.

### Loss Functions

For single-label classification, categorical cross-entropy was used. For multilabel classification, binary cross-entropy was used. To address imbalance, class weights were applied.


(4) $$L=-\sum_{c}w_{c}y_{c}\log({\hat{y}}_{c})$$

where, *w_c_* is inversely proportional to class prevalence.

### Natural Language Generation for Interpretability

#### Prompt Engineering Framework

Integration of GPT-4 for clinical interpretation represents a key innovation in making diagnostic results accessible to nonspecialist health care workers. Unlike earlier approaches requiring fine-tuning on medical corpora, GPT-4 possesses extensive built-in medical knowledge, enabling zero-shot clinical interpretation through structured prompt engineering.

#### Prompting Strategy

A structured prompt template (Textbox 2) was used to generate clinician-oriented interpretations incorporating signal type, predicted label(s), confidence scores, and context. This follows prior evidence that interpretability and uncertainty communication are important for responsible clinical AI deployment.

- Signal type
- Predicted label(s)
- Confidence score(s)
- Requested format: explanation, clinical significance, recommended next steps
- Safety framing: decision support only

Textbox 2. **Structured prompt template**

You are a medical AI assistant helping primary care practitioners interpret biomedical signal analysis results.

Medical signal analysis report:

- Signal Type: {signal_type}
- Classification: {classification}
- Confidence: {confidence:.2%}
- Clinical Context: {context}

Provide a detailed clinical interpretation, including

- Explanation of the finding
- Clinical significance
- Recommended follow-up actions

Keep the response professional, clear, and actionable for health care workers in underserved regions.

#### LLM Integration

An LLM was called through an API to generate structured explanations for predicted outputs. Outputs were framed explicitly as decision support, not autonomous diagnosis, consistent with clinical AI impact guidance and risk considerations about uncertainty communication.

The system leveraged OpenAI’s GPT-4 API for natural language generation, offering several advantages over locally fine-tuned models. These advantages included superior medical knowledge derived from extensive pretraining on medical literature, improved clinical accuracy (4.3/5.0) and clarity (4.6/5.0) based on expert review, elimination of local deployment requirements given GPT-4’s scale (1.7 trillion parameters compared with 345 million for GPT-2-medium), regular model updates provided by OpenAI, and reduced system complexity due to the absence of fine-tuning infrastructure. Trade-offs associated with this approach included the requirement for network connectivity to support API calls, usage-based costs (approximately US $0.02-$0.05 per interpretation), an average generation latency of 1.3 seconds, and data privacy considerations, although API requests were not used for model training. For deployment scenarios with connectivity limitations or strict data locality requirements, future work could explore locally deployable alternatives, such as fine-tuned Llama-3-70B or Mistral-Large models, or an offline fallback using template-based interpretations.

### Experimental Evaluation

#### Experimental Setup

All experiments were conducted on a distributed computing infrastructure comprising 16 nodes, each equipped with 8× NVIDIA A100 (80GB) GPUs. Training used mixed-precision computation (FP16) to optimize memory usage and computational efficiency. Hyperparameter optimization was performed using Bayesian optimization over 50 trials on the validation set.

#### Baseline Methods and Fair Comparison

To ensure rigorous evaluation, we compare the proposed LSTM architecture against 3 established baseline methods under identical experimental conditions.

The baseline methods include the following:

The fair comparison protocols include the following:

- Identical patient-level train/val/test partitions (70/15/15)
- Same modality-specific preprocessing and lead selection
- Same evaluation metrics computed on identical test sets
- Same hardware (NVIDIA Tesla V100 GPU, 32GB RAM)
- Hyperparameter tuning via 5-fold cross-validation on the training set for all methods

#### Evaluation Metrics and Justification

Model performance was evaluated using multiple complementary metrics, each chosen to capture specific aspects of medical diagnostic tasks, as summarized in Table 7. Accuracy was used as a baseline indicator of overall classification correctness. Precision was included to quantify the reliability of positive diagnoses, where false positives could lead to unnecessary interventions. Sensitivity (recall) was critical for identifying pathological conditions, as false negatives can have serious clinical consequences, while specificity was important for ruling out conditions and reducing false alarms in monitoring systems. The *F*_1_-score provided a balance between precision and recall, particularly relevant given class imbalances in biomedical datasets. Finally, the area under the receiver operating characteristic curve (AUROC) assessed discriminative ability across all decision thresholds and is robust to class imbalance, providing a comprehensive measure of model performance.

**Table 7.** Evaluation metrics and rationale.

Metric	Rationale
Accuracy	Overall correctness summary
Sensitivity	Detecting true positives; important for avoiding missed pathology
Specificity	Avoiding false alarms; important for monitoring settings
Precision	Controls false positives; reduces unnecessary interventions
*F*_1_-score	Balances precision and recall in imbalanced data
AUROC^a^	Measures discrimination across thresholds; robust summary statistic

a AUROC: area under the receiver operating characteristic curve.

For multiclass settings, macroaveraging was used to avoid dominance by majority classes.

#### Macroaveraging

All multiclass metrics use macroaveraging rather than microaveraging to ensure fair evaluation across imbalanced classes, preventing performance on common classes from dominating metrics.

#### Statistical Significance

Prior to statistical testing, we assessed normality of performance metric distributions using the Shapiro-Wilk test (*α*=.05). All metrics (accuracy, *F*_1_-score, and AUC) were approximately normally distributed across the 5-fold cross-validation splits (*P*>.05 for all tests), justifying the use of parametric tests. Paired t-tests were conducted to compare model performance across datasets and conditions. Effect sizes were calculated using Cohen *d*, with |*d*|≥0.5 considered meaningful. All statistical analyses were performed using Python 3.9 with SciPy (v1.9.0) and statsmodels (v0.13.0).

Performance comparisons were evaluated using paired *t*-tests with a significance threshold of *P*<.05 across 5 random train or test splits to confirm the statistical significance of observed improvements.

## Results

### Classification Performance

Classification performance was evaluated on held-out test sets, with patient-level splitting applied wherever patient identifiers were available to prevent data leakage and ensure true generalization, consistent with recommended practices for clinical AI evaluation. As summarized in Table 8, the framework demonstrated robust performance across both single-label and multilabel biomedical signal datasets.

**Table 8.** Classification performance metrics on test sets^a^.

Dataset	Accuracy (%)	Sensitivity (%)	Specificity (%)	*F*_1_-score	AUROC^b^
MIT-BIH^c^ Arrhythmia	92.3	89.7	94.1	0.91	0.95
PTB^d^ Diagnostic ECG^e^	94.7	93.2	95.8	0.94	0.97
PTB-XL^f^	88.9	86.4	91.2	0.88	0.93
Chapman-Shaoxing	91.2	88.9	93.1	0.90	0.94
MIMIC-III^g^ Waveforms	89.5	87.1	91.8	0.89	0.92
Sleep-EDF^h^	87.3	84.6	89.7	0.86	0.91
Average	90.7	88.4	92.5	0.90	0.93

a Note: Metrics for multi-label datasets (PTB Diagnostic ECG, PTB-XL, Chapman-Shaoxing) represent macroaveraged performance across all labels. Single-label datasets (MIT-BIH, MIMIC-III, Sleep-EDF) use standard multiclass metrics with macroaveraging.

b AUROC: area under the receiver operating characteristic curve.

c MIT-BIH: Massachusetts Institute of Technology-Beth Israel Hospital.

d PTB: Physikalisch-Technische Bundesanstalt.

e ECG: electrocardiogram.

f XL: extra large.

g MIMIC: Medical Information Mart for Intensive Care.

h EDF: European data format.

Among single-label datasets, the MIT-BIH Arrhythmia dataset achieved 92.3% accuracy and 0.95 AUROC, reflecting well-defined arrhythmia patterns and high-quality expert annotations. The MIMIC-III Waveforms dataset showed 89.5% accuracy and 0.92 AUROC, indicating reliable generalization to diverse ICU waveforms and clinical monitoring scenarios. The Sleep-EDF dataset achieved 87.3% accuracy and 0.91 AUROC, with moderate performance influenced by subjective sleep stage boundaries, interindividual variability, and class imbalance. For multilabel datasets, the PTB Diagnostic ECG dataset achieved 94.7% accuracy and 0.97 AUROC, PTB-XL achieved 88.9% accuracy and 0.93 AUROC, and Chapman-Shaoxing achieved 91.2% accuracy and 0.94 AUROC, with macroaveraged metrics reflecting performance across all co-occurring diagnostic labels. Overall, the system achieved an average accuracy of 90.7% and an AUROC of 0.93 across all datasets, demonstrating consistent and generalizable classification performance while highlighting dataset-specific challenges.

### Baseline Comparison

To validate the effectiveness of the LSTM architecture, performance was compared against 3 baseline methods using identical preprocessing and data splits. The results, summarized in Table 9, show that the proposed LSTM model outperforms traditional machine learning models (support vector machine and random forest) and a 1D CNN baseline in terms of accuracy and *F*_1_-score. Although training time was longer for the LSTM, the gain in classification performance was seen particularly in the *F*_1_-score, which justifies the additional computational cost for clinical applications where diagnostic accuracy was critical. Statistical significance of the improvements was confirmed using paired *t*-tests (*P*<.05).

**Table 9.** Baseline method comparison on Massachusetts Institute of Technology-Beth Israel Hospital Arrhythmia dataset.

Method	Accuracy (%)	*F*_1_-score	Training time (min)	Inference time (ms)
SVM^a^ (RBF^b^)	85.2	0.83	45	12
Random forest	87.6	0.85	32	18
1D CNN^c^	89.8	0.88	78	15
LSTM^d^ (proposed)	92.3	0.91	95	87

a SVM: support vector machine.

b RBF: radial basis function kernel.

c CNN: convolutional neural network.

d LSTM: long short-term memory.

### Computational Performance

System computational efficiency is critical for deployment in resource-constrained environments. As summarized in Table 10, signal preprocessing required 125 ms per sample with 256 MB of memory usage, while LSTM inference took 87 ms and 512 MB of memory. GPT-4-based natural language generation was the most time- and memory-intensive stage, requiring 1.3 seconds and 2.1 GB of memory per interpretation. Overall, the end-to-end pipeline completed in approximately 1.51 seconds, using 2.87 GB of memory, demonstrating the feasibility for real-time clinical decision support in typical computing environments.

**Table 10.** Computational performance benchmarks.

Pipeline stage	Processing time	Memory usage
Signal preprocessing	125 ms	256 MB
LSTM^a^ inference	87 ms	512 MB
GPT-4 generation	1.3 s	2.1 GB
Total end-to-end	1.51 s	2.87 GB

a LSTM: long short-term memory.

Processing time and memory usage were measured across major pipeline components to quantify feasibility for time-sensitive workflows and resource-constrained deployments.

### Quality of Generated Clinical Interpretations

Quality of GPT-4-generated interpretations was evaluated through expert review by 3 board-certified cardiologists using four criteria: clinical accuracy, relevance, clarity, and actionability.

Expert evaluation of GPT-4-generated interpretations demonstrated high quality across multiple criteria, as summarized in Table 11. Clinical accuracy received a score of 4.3 with 92% of criteria met and strong interrater agreement (κ=0.87). Relevance and clarity were similarly high, scoring 4.5 and 4.6 with κ values of 0.91 and 0.89, respectively, while actionability scored 4.2 with 89% of criteria met (κ=0.85). These results indicated that the generated explanations were clinically coherent, contextually relevant, and actionable. The consistently strong interrater agreement across all metrics confirmed the reliability of the generative natural language processing model for clinical decision support.

**Table 11.** Expert assessment of generated interpretations.

Metric	Mean score (1-5)	Interrater agreement (κ)	Criteria met (%)
Clinical accuracy	4.3	0.87	92
Relevance	4.5	0.91	94
Clarity	4.6	0.89	96
Actionability	4.2	0.85	89

## Discussion

### Performance Analysis and Cross-Dataset Interpretation

The experimental results demonstrate that the proposed pipeline achieves robust performance across multiple biomedical signal modalities using a consistent single-lead, single-label approach. The 2-layer LSTM architecture effectively captures temporal dependencies in physiological signals, while integration with GPT-4 provides interpretable outputs suitable for clinical decision-making. Performance variation across datasets reflects differences in signal characteristics, annotation quality, and clinical complexity. For single-label tasks, the model achieved the highest performance on MIT-BIH Arrhythmia (92.3% accuracy, 0.95 AUROC), benefiting from a high signal-to-noise ratio, gold-standard expert annotations, and sufficient sample representation for majority classes, with class weighting compensating for rare arrhythmias. Misclassifications primarily occurred between normal and right bundle branch block beats or fusion and premature ventricular complex beats, consistent with known interrater disagreement. Sleep-EDF showed moderate performance (87.3% accuracy, 0.91 AUROC), influenced by subjective sleep stage boundaries, low EEG amplitude, inter-individual variability, and class imbalance, with most errors occurring between adjacent stages (N1↔N2, N2↔N3, and wake↔N1). MIMIC-III performance (89.5% accuracy, 0.92 AUROC) reflects clinical diversity, prevalence of artifacts, variable sampling rates, and label noise, with misclassifications observed between normal sinus rhythm and sinus tachycardia, and between atrial fibrillation and atrial flutter.

We adopted a single-label (mutually exclusive) classification formulation rather than multilabel prediction for several reasons aligned with the resource-constrained deployment context. First, single-label classification simplifies clinical decision-making by providing a definitive primary diagnosis, which is more actionable for nonspecialist providers in low-resource settings. Second, the selected datasets (MIT-BIH, PTB, and CHB-MIT) were originally curated with mutually exclusive diagnostic categories, making single-label formulation more appropriate. Third, multi-label approaches substantially increase computational and interpretative complexity, requiring more sophisticated threshold selection and potentially generating conflicting diagnoses. For future work in contexts where co-occurring conditions are common (eg, concurrent arrhythmias), multilabel formulations with hierarchical class structures would be valuable extensions.

In comparison, multilabel datasets require independent probability estimation for each diagnosis via sigmoid activation. PTB Diagnostic ECG achieved high performance (94.7%) due to its binary classification structure, whereas PTB-XL (88.9%) reflects the challenge of predicting 71 simultaneous diagnostic categories with class imbalance. Chapman-Shaoxing performed moderately well (91.2%), demonstrating the model’s ability to handle co-occurring rhythm categories. Overall, single-label tasks benefit from softmax normalization and are suitable for point-of-care scenarios where a primary diagnosis is needed, while multilabel tasks enable comprehensive diagnostic assessments in better-resourced clinical settings.

### Cross-Dataset Generalization and Preprocessing Impact

Modality-specific preprocessing was essential for consistent performance. ECG signals were filtered at 0.5‐50 Hz to preserve QRS morphology, while EEG signals were filtered at 0.5‐30 Hz to retain physiologically relevant sleep frequency bands (Table 2). Ablation studies indicate that uniform filtering across modalities reduces average accuracy by 4.1%, confirming the importance of adaptive preprocessing. Single-lead selection (Table 4) and fixed-sample segmentation (Table 3) ensured reproducible input dimensions, supported point-of-care feasibility, and prevented data leakage via patient-level splitting.

### Clinical Implications

GPT-4-generated natural language interpretations bridge the gap between raw signal classification and actionable clinical insights, enabling non-specialist health care workers to understand diagnostic reasoning. Cloud-based deployment considerations include connectivity, API costs, and regulatory compliance. The system’s interpretability and alignment with portable ECG devices (eg, AliveCor) support deployment in resource-limited environments.

### Comparison With State-of-the-Art

Compared with previous works, our system achieves competitive arrhythmia detection (92.3% accuracy vs 94.2%) [25] and AUROC (0.95 vs 0.96) [26], with the added advantage of single-lead compatibility and natural language output. Unlike text-only models such as BioBERT or ClinicalBERT, this pipeline integrates physiological signals with generative AI for end-to-end interpretability.

### Limitations and Future Work

The study represents a technical proof-of-concept validated on retrospective public datasets only. The system has not been deployed in clinical settings, validated prospectively, or tested for patient outcome improvements. Limitations include single-label focus, single-lead restriction, potential dataset bias, cloud-based computational requirements, and limited dataset diversity. Future work includes prospective IRB-approved clinical pilots, multilead and multilabel extensions, multimodal integration, federated learning for privacy preservation, uncertainty quantification, edge deployment optimization, and cost reduction through alternative LLMs. Regulatory approval and usability testing will be essential before clinical deployment.

Several important limitations warrant discussion. First, this proof-of-concept was evaluated exclusively on curated public datasets that may not reflect the noise characteristics, artifact levels, and signal quality typical of real-world field deployments in resource-limited settings. Robustness to motion artifacts, electrode contact issues, and electrical interference remains untested. Second, our framework relies on cloud-based GPT-4 API calls, raising concerns about (1) data privacy and HIPAA compliance when transmitting patient signals, (2) dependence on stable internet connectivity, and (3) potential API cost barriers for sustained deployment. Alternative approaches using locally deployed open-source LLMs (eg, Llama-2 and Mistral) should be explored, though preliminary tests suggest current open models produce less clinically coherent interpretations. Third, we have not evaluated real-time performance constraints or edge-device deployment feasibility. The LSTM inference time (approximately 50 ms per signal on GPU) is acceptable, but resource requirements on low-power devices (eg, Raspberry Pi or mobile platforms) are unknown. Fourth, the PhysioNet datasets used may not adequately represent demographic diversity (age, sex, race, and comorbidities) or rare conditions, potentially limiting generalizability and introducing bias. Finally, while expert evaluation was promising (mean rating 4.2/5, SD 0.57), it was limited to 150 interpretations and did not assess long-term clinical impact or actual provider acceptance in practice settings.

This work presents a technical proof-of-concept and has not been deployed in clinical settings or evaluated using prospective patient data. The system remains a research prototype and would require extensive prospective validation across diverse patient populations, formal clinical trials with IRB approval prior to any clinical use, regulatory clearance (eg, Food and Drug Administration 510(k) or equivalent) for diagnostic applications, and ethics review for telemedicine deployment in underserved regions.

Generalization of model performance beyond PhysioNet benchmarks remains uncertain, particularly in real-world environments with differing patient populations and signal acquisition equipment. There is a risk of automation bias, whereby health care workers may overrely on AI-generated outputs, underscoring the need for interface designs that encourage critical evaluation. Future deployments must incorporate end-to-end encryption and secure data handling mechanisms to protect patient privacy. Liability considerations related to AI-assisted misdiagnosis remain unresolved and will require clearly defined legal and regulatory frameworks before clinical use.

### Conclusion

Important caveats should be noted regarding near-term clinical deployment. This proof of concept demonstrates technical feasibility on retrospective public datasets but has not been validated in prospective real-world settings. Critical next steps include (1) prospective pilot studies in low-resource primary care clinics to assess real-world performance and provider acceptance; (2) testing on portable, low-power edge devices to confirm computational feasibility without cloud infrastructure; (3) evaluation with locally deployed open-source LLMs to eliminate API dependencies and privacy concerns; and (4) assessment of performance on signals with realistic noise and artifact levels. Only after these validations can clinical deployment be responsibly considered.

This work presents a comprehensive technical framework integrating LSTM-based biomedical signal classification with GPT-4-generated natural language interpretation, designed for deployment in resource-limited and remote settings. The framework’s key contributions include a single-lead selection strategy (Table 4) for consistent input dimensionality and alignment with point-of-care devices, modality-specific preprocessing (Table 2) that preserves diagnostically relevant features while removing artifacts, and a unified architecture supporting both single-label (softmax) and multilabel (sigmoid) formulations, enabling applicability across diverse diagnostic scenarios. Patient-level data splitting ensures true generalization without leakage, while robust classification performance was demonstrated across 6 datasets, including MIT-BIH (92.3%), PTB Diagnostic (94.7%), PTB-XL (88.9%), Chapman-Shaoxing (91.2%), MIMIC-III (89.5%), and Sleep-EDF (87.3%). GPT-4-generated explanations achieved high clinical accuracy (4.3/5.0) and clarity (4.6/5.0) as assessed by expert reviewers (Table 11), highlighting the framework’s practical clinical utility for interpretable AI-assisted decision support. The open-source implementation with Representational State Transfer API ensures transparency, reproducibility, and community validation. While prospective clinical deployment and real-world validation remain future steps, this framework provides a robust, methodologically transparent baseline for AI-driven diagnostics and supports equitable access to remote diagnostic tools across diverse health care settings.

## Abbreviations

AI: artificial intelligence
API: application programming interface
AUROC: area under the receiver operating characteristic curve
CHB-MIT: Children's Hospital Boston-Massachusetts Institute of Technology
CNN: convolutional neural network
ECG: electrocardiogram
EDF: European data format
EEG: electroencephalogram
HIPAA: Health Insurance Portability and Accountability Act
ICU: intensive care unit
IRB: Institutional Review Board
LLM: large language model
LSTM: long short-term memory
MIH-BIH: Massachusetts Institute of Technology-Beth Israel Hospital
MIMIC: Medical Information Mart for Intensive Care
PTB: Physikalisch-Technische Bundesanstalt
PTB-XL: Physikalisch-Technischen Bundesanstalt-extra large
ReLU: rectified linear unit
